# 0590. Impact of arterial tone changes on dynamic arterial elastance and the arterial pressure response to fluid administration

**DOI:** 10.1186/2197-425X-2-S1-P35

**Published:** 2014-09-26

**Authors:** MI Monge García, M Gracia Romero, P Guijo González, A Gil Cano, J Mesquida, R Andrew, RM Grounds, M Cecconi

**Affiliations:** St. George's Healthcare NHS Trust and St George's University of London, Department of Intensive Care Medicine, London, UK; Hospital SAS de Jerez, Servicio de Cuidados Intensivos y Urgencias, Jerez de la Frontera, Spain; Universitat Autónoma de Barcelona, Consorci Sanitari Universitari Parc Tauli, Barcelona, Spain

## Introduction

Dynamic arterial elastance (Ea_dyn_), the relationship between pulse pressure variation (PPV) and stroke volume variation (SVV), has been suggested as a functional assessment of arterial load for predicting the arterial pressure response after volume expansion (VE)^1^. Although changes in Ea_dyn_ have been related with variations in arterial load^2^, the effect of acute arterial tone changes on Ea_dyn_ and the impact on its performance for predicting the arterial pressure response after VE has not yet been determined.

## Objective

To evaluate the effect of acute arterial tone changes on Ea_dyn_ and the influence on its performance for predicting arterial changes after fluid administration.

## Methods

12 anesthetized and mechanically ventilated rabbits. Arterial tone changes were induced by phenylephrine (PHENY) infusion on 6 animals (HighMAP group) and by sodium nitroprusside (SNP) on the other 6 animals (LowMAP group), until reach a 50% of change on mean arterial pressure (MAP) from its baseline value. A volume challenge (10 mL/Kg) was then performed on all animals. Animals were monitored with an indwelling femoral arterial catheter and an esophageal Doppler (CardioQ-Combi). Arterial load was assess by the systemic vascular resistance, net arterial compliance and effective arterial elastance. Ea_dyn_ was calculated as the simultaneous ratio between PPV and SVV obtained from the Doppler monitor.

## Results

**Figure 1:**
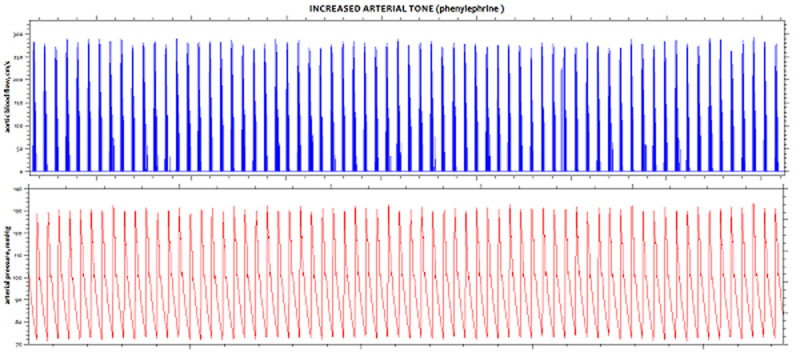
SVV and PPV example on HighMAP group.

**Figure 2:**
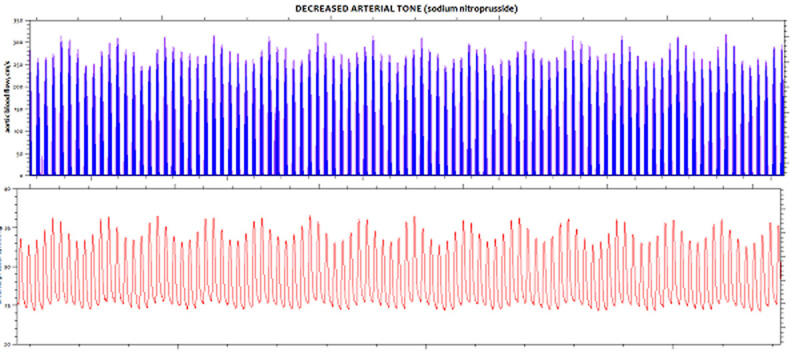
SVV and PPV example on LowMAP group.

At baseline, Ea_dyn_ and other arterial load parameters were similar on both groups. In the LowMAP group, SNP significantly decreased arterial load, reduced MAP by 44%, and consistently increased Eadyn by 75% (Figure 1). In the HighMAP group, PHENY increased arterial load, raised MAP by 58%, and significantly reduced Ea_dyn_ by 41% (Fig. 1 and 2). Overall, VE increased cardiac output by 10%, stroke volume by 21% and MAP by 15%, and decreased Ea_dyn_ from 1.08 ± 0.67 to 0.88 ± 0.45 (Fig.1). There was a significant relationship between Ea_dyn_ after arterial tone changes and increases in all components of arterial pressure after VE: systolic (R^2^=0.89), diastolic (R^2^=0.41), mean arterial (R^2^=0.61) and pulse pressure (R^2^=0.67), respectively. Animals with a MAP increase ≥ 10% after VE had a higher preinfusion Ea_dyn_ value (1.54 ± 0.49 vs. 0.46 ± 0.15; P < 0.001).

**Table 1:** Comparison of arterial load parameters during different experimental conditions in HighMAP abnd LowMAP groups (n=6 on both experimental arms).

	Baseline	After change in arterial pressure	Postinfusion	P value^a^
**EA**_**dyn**_				
HighMAP	0.92 ± 0.12	0.52 ± 0.23*	0.49 ±.0.18	<0.001
LowMAP	0.91 ± 0.16	1.64 ± 0.44*	1.26 ± 0.22^†^*	
**Ea, cmHg/mL**				
HighMAP	3.99 ± 0.93	5.42 ± 1.46*	4.45 ± 1.11^†^	<0.001
LowMAP	4.50 ± 1.44	1.80 ± 0.63*	1.52 ± 0.50^†*^	
**C, mL/cmHg**				
HighMAP	0.66 ± 0.13	0.41 ± 0.10*	0.48 ± 0.08^†*^	<0.001
LowMAP	0.53 ± 0.16	1.92 ± 0.86*	1.83 ± 0.58*	
**TVSR, MPa's/m** ^**3**^				
HighMAP	1514 ± 453	3157 ± 1299*	3372 ± 1306*	<0.001
LowMAP	1814 ± 585	709 ± 215*	720 ± 218*	

C: new arterial compliance; Ea: effective arterial elastance; Eadyn: dynamic arterial elastance; TSVR: total systemic vascular resistance. Data are presented as mean ± standard deviation. Data normally distributed according to Kolomogorov-Smirnov test. Note that pressure units are expressed as cmHg for convenience. *p<0.05 vs baseline. ^†^p<0.05 vs. change in arterial pressure. *p value refers to ANOVA test for time and group interaction

## Conclusions

In this experimental settings acute modifications on arterial tone induced significant changes on Ea_dyn_: arterial vasodilation increased Ea_dyn_, whereas vasoconstriction decreased it. Nevertheless, preinfusion Ea_dyn_ still determined the arterial pressure response after volume administration.
